# Co-cultivation of *Aspergillus nidulans* Recombinant Strains Produces an Enzymatic Cocktail as Alternative to Alkaline Sugarcane Bagasse Pretreatment

**DOI:** 10.3389/fmicb.2016.00583

**Published:** 2016-04-28

**Authors:** Matheus S. Lima, André R. de L. Damasio, Paula M. Crnkovic, Marcelo R. Pinto, Ana M. da Silva, Jean C. R. da Silva, Fernando Segato, Rosymar C. de Lucas, João A. Jorge, Maria de L. T. de M. Polizeli

**Affiliations:** ^1^Department of Biology, Faculty of Philosophy, Sciences and Letters of Ribeirão Preto, University of São PauloSão Paulo, Brazil; ^2^Department of Biochemistry and Tissue Biology, Institute of Biology, University of CampinasSão Paulo, Brazil; ^3^Department of Mechanical Engineering, University of São PauloSão Paulo, Brazil; ^4^Laboratory of Biopathology and Molecular Biology, Uberaba UniversityUberaba, Brazil; ^5^Federal University of Campina GrandeCuite, Brazil; ^6^Federal Institute of Education, Science and Technology of São PauloSão Paulo, Brazil; ^7^Department of Biotechnology, Engineering School of Lorena, University of São PauloSão Paulo, Brazil; ^8^Department of Biochemistry and Immunology, Ribeirão Preto Medical School, University of São PauloSão Paulo, Brazil

**Keywords:** *Aspergillus nidulans*, co-cultivation, thermogravimetric analysis, differential thermal analysis, NaOH pretreatment, enzymatic pretreatment, sugarcane bagasse, enzymatic cocktail

## Abstract

Plant materials represent a strategic energy source because they can give rise to sustainable biofuels through the fermentation of their carbohydrates. A clear example of a plant-derived biofuel resource is the sugar cane bagasse exhibiting 60–80% of fermentable sugars in its composition. However, the current methods of plant bioconversion employ severe and harmful chemical/physical pretreatments raising biofuel cost production and environmental degradation. Replacing these methods with co-cultivated enzymatic cocktails is an alternative. Here we propose a pretreatment for sugarcane bagasse using a multi-enzymatic cocktail from the co-cultivation of four *Aspergillus nidulans* recombinant strains. The co-cultivation resulted in the simultaneous production of GH51 arabinofuranosidase (AbfA), GH11 endo-1,4-xylanase (XlnA), GH43 endo-1,5-arabinanase (AbnA) and GH12 xyloglucan specific endo-β-1,4-glucanase (XegA). This core set of recombinant enzymes was more efficient than the alternative alkaline method in maintaining the cellulose integrity and exposing this cellulose to the following saccharification process. Thermogravimetric and differential thermal analysis revealed residual byproducts on the alkali pretreated biomass, which were not found in the enzymatic pretreatment. Therefore, the enzymatic pretreatment was residue-free and seemed to be more efficient than the applied alkaline method, which makes it suitable for bioethanol production.

## Introduction

Plant biomass stores large amounts of complex carbohydrates (Fujii et al., 2009) which can be converted to fermentable sugars and consequently to products with high added value. Sugarcane bagasse is a robust source of this biomass. Brazil, for instance, produced about 632 million tons of sugarcane in 2014/2015^1^, wherein 25% of this quantity was bagasse, which contains 60–80 % of carbohydrates in its composition (Betancur and Pereira, 2010).

However, biomass carbohydrates (cellulose and hemi cellulose) together with lignin are strongly organized, chemically and physically, through chemical bonds such as non-covalent forces and covalent cross-linkages (Pérez et al., 2002). This structure acts as skin and backbone in the plant, representing a barrier for the whole bioconversion process (Dashtban et al., 2009). Therefore, biomass pretreatment is a requisite.

The pretreatment can be performed through different methods such as diluted acid, liquid hot water, AFEX, alkali and organosolv processes (Balan et al., 2009; Kim et al., 2009; Zhao et al., 2009; Yang and Wyman, 2009; Sharma et al., 2011). Each method acts in a particular way; either modifying cellulose crystallinity or removing hemicellulose and lignin from the plant cell wall matrix (Banerjee et al., 2010a). But, the ideal pretreatment should also reduce carbohydrate loss, the amount of enzyme inhibitors and toxic compounds normally generated during these chemical/physical processes (Rezende et al., 2011). In that sense, enzymes produced by microorganisms such as, fungi and bacteria are now replacing chemicals, originating a less harmful alternative for biomass pretreatment (Almeida et al., 2007; Meyer et al., 2009). Filamentous fungi, notably *Aspergillii* and *Trichoderma*, are attractive because they are traditionally important producers of commercial cellulases and xylanases and widely applied in the production of recombinant enzymes (Polizeli et al., 2005; Muthezhilan et al., 2007; Kumar et al., 2008; Visser et al., 2011). However, the available commercial enzymes preparations for biomass pretreatment are limited in number and composition, being generally optimized for the hydrolysis of corn stover and some grasses such as switchgrass and *Miscanthus* sp. (Banerjee et al., 2010b).

Co-culture of two or more organisms, whether recombinants or not, addresses this problem by generating a diverse and efficient enzymatic cocktail. In natural environments such as forest soils, compost piles and mammalian intestines, there are endless examples of coexistent microorganisms, mutualism in many cases (Qi-he et al., 2011) illustrating the importance of cooperation for achieving a synergistic (and optimized) enzymatic action (Dong et al., 2012). In fact, many food and pharmaceutical production lines already employ co-cultivation of microorganisms (Qi-he et al., 2011).

The aim of the present work was to co-cultivate *Aspergillus nidulans* recombinant strains for the simultaneous production of four glycoside hydrolases from families 11, 12, 43, and 51. Once successful, the enzymatic cocktail resulted from the co-culture was used as a pretreatment for sugarcane bagasse in alternative to alkali pretreatment. Finally, SEM and thermal analysis (TG/DTG and DTA) were applied to assess how each pretreatment (enzymatic vs. alkali) modified the sugarcane bagasse, in order to posterior incubation with cellulases and reducing sugar production.

## Materials and Methods

### *Aspergillus* Cultivation

*Aspergillus nidulans* A773 was kindly provided by Dr. Rolf Prade, from Department of Microbiology and Molecular Genetics, Oklahoma State University, USA, which came from the FGSC (St. Louis, MO, USA). Standard *A. nidulans* MM and general cultivation techniques were based on Pontecorvo et al. (1953) and Clutterbuck (1996).

### Construction of *pEXPYR*-Client Proteins Plasmids

PCR-amplified gene-fragments were digested with NotI and XbaI, ligated onto NotI/XbaI digested pEXPYR plasmid with T4-fast ligase (Promega, WI) and transformed into Ca^+^ competent *Escherichia coli* TOP 10F’ competent cells (Invitrogen, CA; Sambrook et al., 1989). Plasmids with the correct insert size DNA were fully sequenced at Oklahoma State University Core Facility and clones with the correct DNA sequence were used for transformation.

Recombinant pEXPYR plasmid was introduced through integrative transformation into *A. nidulans* strain FGSC A773 (pyrG, pyroA) genome (Segato et al., 2012) and recombinants selected on MM supplemented with 1 mM pyridoxine and 100 μg/mL zeocin. Five pyrG+, zeocin resistant transformants, were grown on plates containing 10 mL MM, pyridoxine and 5% maltose, for 48 h at 37°C. Secretion of client proteins in the medium was analyzed by SDS-PAGE and one transformant for each enzyme was used for further investigation.

### Co-cultivation of *A. nidulans* Recombinant Strains

A concentration of 10^7^–10^8^ spores/mL of four *A. nidulans* recombinant strains (Damasio et al., 2011, 2012a,b,c) were inoculated into liquid MM supplemented with 5% maltose. Inoculated media were distributed onto dishes (10 mL in 60 mm, 20 mL in 150 mm Petri-dishes and 500 mL onto cafeteria trays) and incubated at 37°C for 2–3 days. Mycelia were separated by filtration and the filtrated medium was centrifuged at 10,000 ×*g* for 10 min prior to concentration by ultra-filtration using 10 kDa cutoff Amicon^®^ (Millipore). The total amount of proteins were quantified by Bradford method (Bradford, 1976) and the secretome analyzed by SDS PAGE (Shapiro et al., 1967). The heterologous proteins ratio (%) was estimated using the ImageJ Software based on a standard gray scale (Research Services Branch, National Institute of Mental Health, Bethesda, MD, USA, wayne@codon.nih.gov).

### Chemical and Enzymatic Pretreatments

**FIGURE 1:**
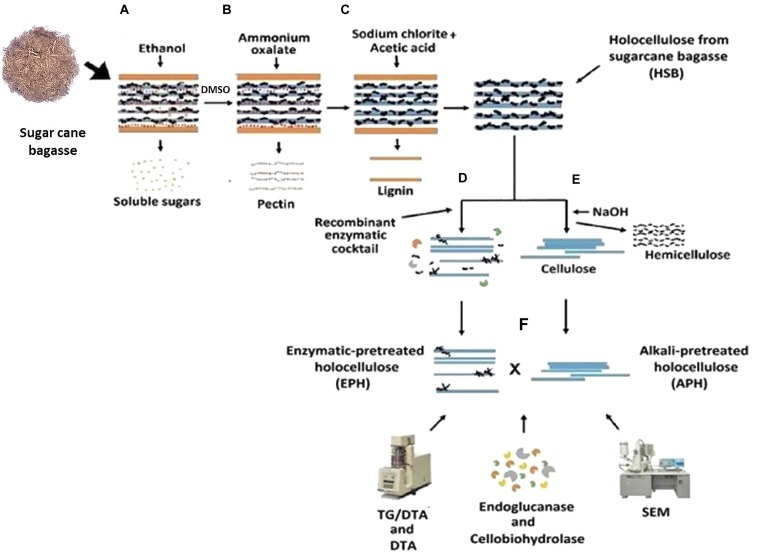
**Experimental design for the sugarcane bagasse chemical fractionation.** Sugarcane bagasse was chemically fractionated having its soluble sugars, starch, pectin and lignin removed **(A–C)**. Holocellulose from sugarcane bagasse (HSB) was further pretreated with the recombinant enzymatic cocktail **(D)** or with 0.1, 1, and 4 M NaOH solutions **(E)**. The two fractions of residual cellulose were hydrolyzed by endoglucanase and cellobiohydrolase of *Scytalidium thermophilum* and analyzed using TG and DTA, and SEM **(F)**.

Chemical pretreatment of sugar cane bagasse: the bagasse *in natura* was treated according to a plant cell wall chemical fractionation protocol modified from Carpita (1983), Gorshkova et al. (1996), and Moraes et al. (2001) (**Figure 1**). One gram of dried and pulverized sugarcane bagasse was incubated with 20 mL of 80% ethanol, at 80°C, for 20 min for the elimination of soluble sugars, and this procedure was repeated six times. The sample was centrifuged for 15 min (10,000 ×*g*) and the pellet was washed with 20 mL of distilled H_2_O and dried at 60°C, overnight (**Figure 1A**). After that, the material was incubated with 20 mL of 90% DMSO, for 24 h, at 90°C for starch removal. The following pectin removal was carried out by the incubation of the residual biomass with 20 mL of 0.5% ammonium oxalate, pH 7.0, at 80°C, for 3 h (**Figure 1B**). Finally, the delignification was performed by incubating the remaining bagasse with 20 mL of 0.5 M sodium chlorite/acetic acid solution, at 65°C for 1 h (**Figure 1C**). This partially fractionated biomass was named holocellulose from sugarcane bagasse (HSB).

Then, the HSB was split in two groups: the APH – Alkali Pretreated Holocellulose – and the EPH – Enzymatic Pretreated Holocellulose (EPH; **Figures 1D–F**):

Enzymatic Pretreated Holocellulose: 500 mg of EPH was hydrolyzed with 10 mL of buffered recombinant enzymatic cocktail (6 mL of the co-cultivation filtrate containing: AbfA, XlnA, AbnA and xyloglucan specific endo-β-1,4-glucanase, and 4 mL of 50 mM ammonium acetate buffer, pH 5.0), for 3 h at 50°C under stirring (**Figure 1D**). Hydrolysis was monitored by measuring the amount of released reducing sugar using DNS (Miller, 1959). ANOVA by one-way ANOVA and Tukey’s multiple comparison tests were performed to verify the relation between time and hydrolysis rate.

Alkali pretreated holocellulose (**Figure 1E**) was treated following the final step of the plant cell wall chemical fractionation: 500 mg were sequentially incubated in 20 mL of three NaOH solutions (0.1, 1.0, and 4.0 M) always in combination with 0.1 M sodium borohydride during 1 h for each solution at room temperature. The hemicellulose released from each NaOH incubation was quantified by phenol-sulfuric acid method (Dubois et al., 1956). Samples were exhaustively washed with distilled water.

### Scanning Electron Microscopy (SEM)

The APH and the EPH were characterized using SEM. Bagasse samples were washed in water, dried at 45°C overnight and sprayed in micro crusher type Willey (Tecnal) to 30 mesh. Samples were mounted on aluminum stubs with double-sided carbon tape and sputter-coated with gold-palladium. All images were acquired by SEM (Zeiss EVO50) operating at 20 kV of the acceleration voltage and with 500× of magnification settings.

### Thermogravimetric Analysis (TG) and Differential Thermal Analysis (DTA)

Thermogravimetric and differential thermal analysis (TG/DTG and DTA) were performed for *in natura* bagasse and both pretreatments (enzymatic and NaOH) by using Shimadzu analyzers – TGA – 50H and DTA-51 models for TG/DTG and DTA, respectively. A sample mass of 10.0 ± 0.5 mg was placed in an alumina crucible and heated from room temperature up to 800°C at a heating rate of 10°C min^–1^. Experiments were performed under an air atmosphere at flow rate of 100 mL min^–1^.

### Cellulose Exposition Estimation Using Purified *Scytalidium thermophilum* Cellulases

Alkali pretreated holocellulose and EPH were compared based on how much their residual cellulose was exposed ( CEE). In order to estimate that, APH and EPH were hydrolysed by pure cellulases from the thermophilic fungus *Scytalidium thermophilum* (CBS 619.91), which was obtained as described by Silva et al. (2013). The best pretreatment would expose most of the cellulose to the purified cellulases without inhibiting the enzymes action. Consequently, the hydrolysis would be more efficient, releasing more reducing sugar. Hydrolysis reactions were carried out with endoglucanase (2.7 U/g bagasse) and cellobiohydrolase (5.86 U/g bagasse) in sodium acetate buffer 50 mM, pH 5.0, containing substrates at 1% (w/v) and maintained for 24 h at 50°C under stirring. The amount of the released reducing sugars was measured by DNS method (Miller, 1959). ANOVA by one-way ANOVA and Tukey’s multiple comparison tests was performed to verify the significant statistical differences in the hydrolysis rate of each substrate.

### Determination of Enzymatic Assays

Endoglucanase (CMCase) activity was assayed by incubating 0.5 mL of enzyme preparation with 0.5 mL of 2% carboxymethylcellulose, CMC (Sigma^®^) dissolved in 100 mmol/L sodium acetate buffer, pH 4.0. Exoglucanase (Avicelase) activity was determined by using 2% microcrystalline cellulose, Avicel (Sigma^®^) as substrate, suspended in 100 mmol/L sodium acetate buffer, pH 6.0. Filter paper activity (FPase) was determined in 100 mmol/L sodium acetate buffer (pH 5.0) using a strip (10 mm × 30 mm) of Whatman n°1 filter paper as substrate. The reactions were carried out at 55°C for 30 min, and enzyme preparations were conveniently diluted to assure the estimation of initial velocities. The reducing sugars released were determined by DNS method (Miller, 1959). One enzyme unit (U) was defined as the amount of enzyme that releases 1 μmol of reducing sugars per min under standard assay conditions.

Endo-1,4-beta-xylanase, endo-1,5-α-L-arabinanase, α-L- AbfA, xyloglucan endo-beta-1,4-glucanase activity were also quantified by the evaluation of reducing sugars release by DNS method (Miller, 1959). The reaction was performed using 0.05 mL substrate (1% w/v), xylan from birchwood (Sigma^®^), debranched arabinan, arabinan from sugar beet and xyloglucan from tamarind (Megazyme^®^), respectively, in 50 mM ammonium acetate buffer, pH 5.0 and 0.05 mL enzyme solution, followed by the incubation at 60°C in a water bath for 15 min. The reaction was stopped by adding 0.1 mL of DNS, followed by boiling for 5 min. One enzyme unit was defined as the amount of enzyme which releases 1 μmol of product per minute, under the assay conditions.

### Reproducibility of the Results

All experiments (when applicable) were independently performed in triplicates exhibiting consistent results.

## Results

### Co-cultivation of *A. nidulans* Recombinant Strains

SDS-PAGE gel presents all recombinant hemicellulases successfully expressed individually; GH51 AbfA (88.6 kDa), GH11 XlnA (36 kDa), GH43 arabinanase (AbnA; 34 kDa) and GH12 xyloglucan-specific endoglucanase (XegA; 28 kDa) demonstrated at lanes 1, 2, 3, and 4, respectively.

**FIGURE 2:**
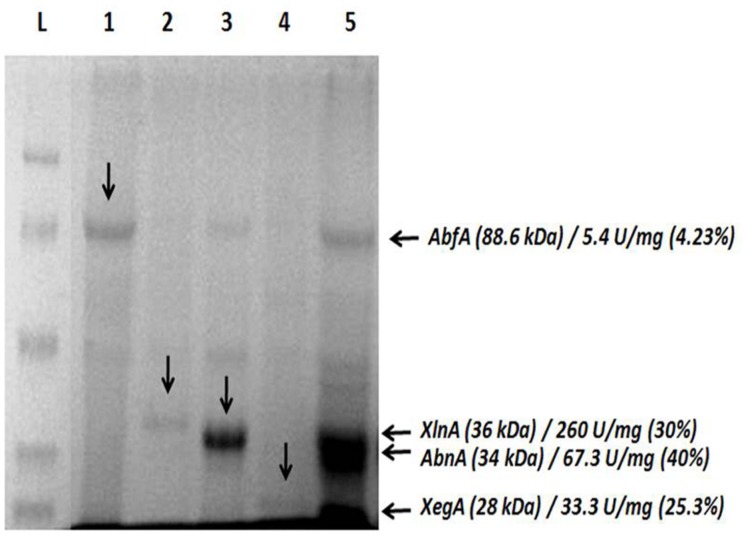
**Simultaneous secretion of arabinofuranosidase, endo-xylanase, arabinanase, and xyloglucanase by co-cultivation of *Aspergillus nidulans* recombinant strains.** (L) ladder; (1) arabinofuranosidase (AbfA, AEQ94263); (2) endo-xylanase (XlnA, AEV23009) (3) arabinanase (AbnA, AEV23010); (4) xyloglucanase (XegA, AEV23011); (5) co-secretion. The amount (%) of each recombinant protein was estimated based on a calibrated grayscale by ImageJ.

In addition to that, the co-cultivation approach, lane 5, resulted in simultaneous production of these enzymes, AbfA, XlnA, arabinanase and xyloglucan-specific endoglucanase with specific activity (U/mg) of 5.4, 260, 67.3, and 33.3, respectively. The ratio (%) of each target enzyme relative to total secretome was also estimated (**Figure 2**). No residual cellulase activity was detected (data not shown).

**FIGURE 3:**
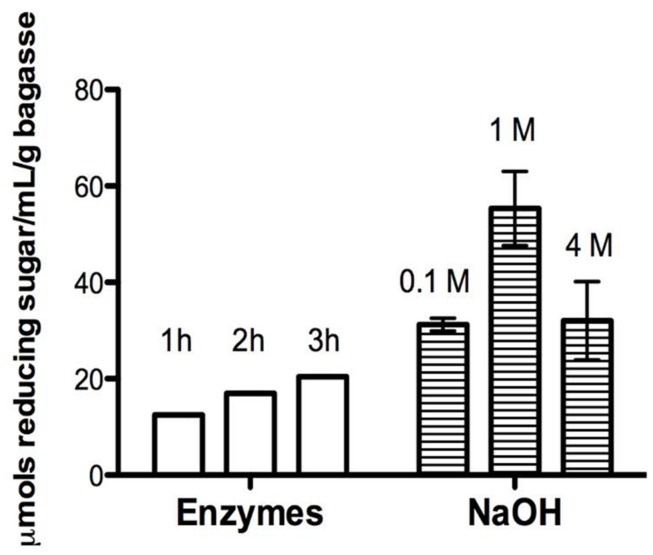
**Time course hydrolysis of holocellulose from sugarcane bagasse (HSB).** Reducing sugars quantified from 1 to 3 h of enzymatic or alkaline treatment by DNS method (Miller, 1959). The enzymatic assays were carried out using a buffered enzymatic solution (co-cultivation filtrate cointaning AbfA, XlnA, AbnA, and XegA, and 50 mM ammonium acetate buffer, pH 5.0) at 50°C, under stirring. Alkaline treatment was carried out at room temperature consecutively using 0.1, 1, and 4 M NaOH solutions always in combination with 0.1 M sodium borohydride.

In order to test the catalytic effect of this core of enzymes over a recalcitrant biomass, a hydrolysis assay was performed. Thus, a significant hydrolysis of sugarcane bagasse (75% of sugar release in 2 h treatment) was achieved, what is an evidence that the recombinant enzymatic cocktail was able to deconstruct hemicellulose (*P* < 0.0001; **Figure 3**) at mild conditions (50°C, pH 5.0).

### Morphological and Structural Changes in APH and EPH

**FIGURE 4:**
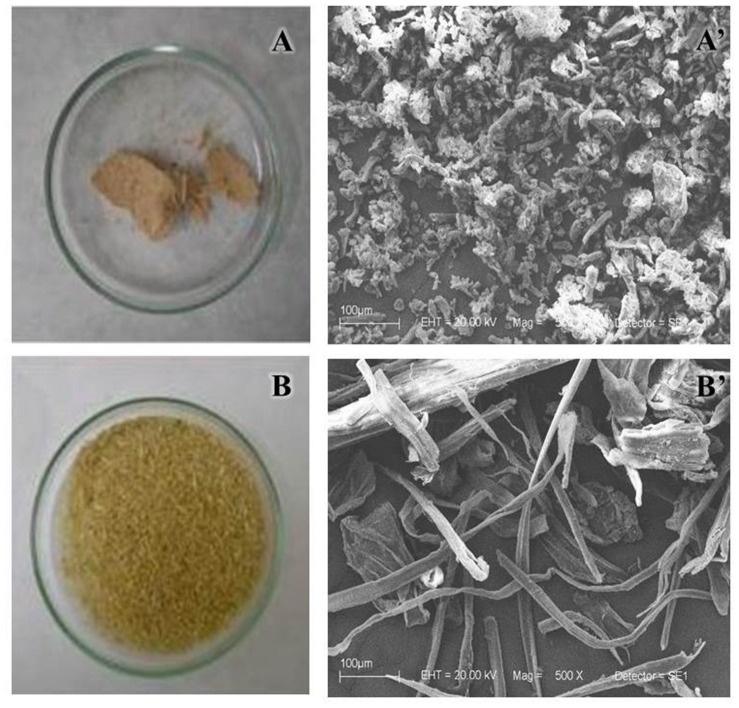
**Overview of pretreated bagasses.** SEM (500×) of the bagasse pretreated with NaOH **(A,A’)** and with enzymatic cocktail **(B,B’)**. After the pretreatments, the samples were washed in water and dried overnight at 45°C.

NaOH method strongly removed the hemicellulose (**Figure 3**), fully deconstructing the bagasse (**Figures 4A,A’**). The result was an APH with a morphology characterized by a non-fibrous appearance. On the other hand, the EPH was only partially hydrolyzed by the recombinant cocktail having its hemicellulose gently removed and remaining its cellulose fibers essentially intact (**Figures 4B,B’**).

### Thermogravimetric and Differential Thermal Analyses (TG/DTA)

**FIGURE 5:**
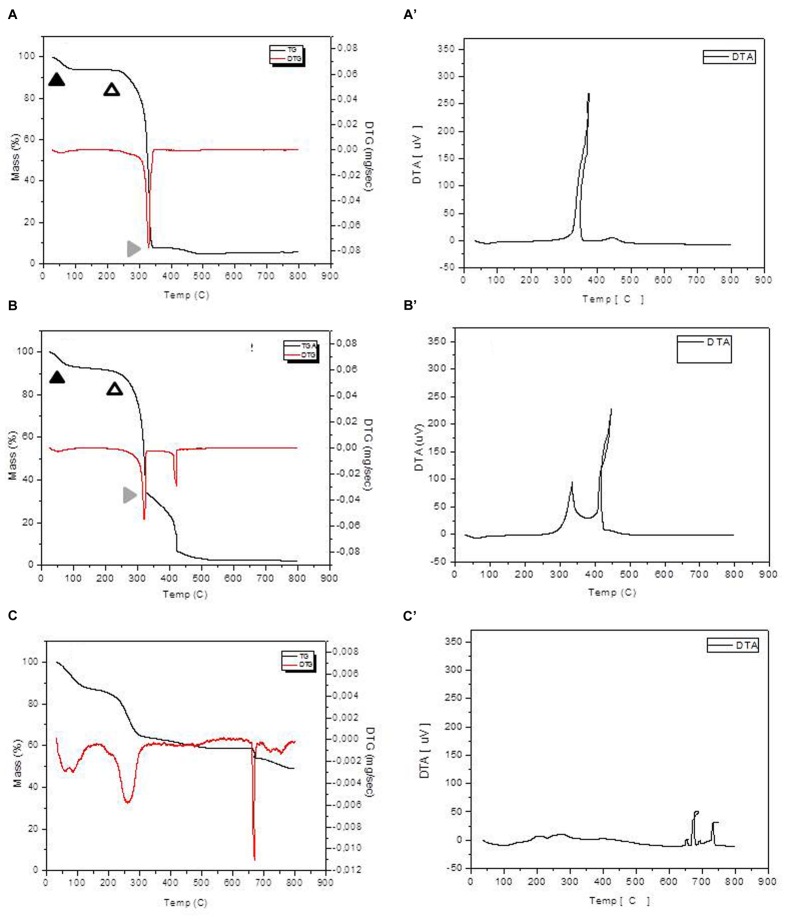
**Thermogravimetric analysis/DTG and DTA analysis for the *in natura* and pretreated bagasse samples.** The *in natura* bagasse **(A)** and the enzymatic pretreated bagasse **(B)** exhibited three clearly distinct mass loss events: moisture release, hemicellulose, cellulose, and lignin decomposition and final lignin degradation represented by full black arrowhead, empty arrowhead and gray arrowhead, respectively. The NaOH pretreated bagasse **(C)** exhibited five mass loss events hardly distinguishable. **A** = In natura bagasse TG, **A’** = in natura bagasse DTG; **B** = EPH TG, **B’** = EPH DTG; **C** = APH TG, **C’** = APH DTG.

The thermo gravimetric (TG) and the first derivative curve (DTG) profiles of *in natura* bagasse and EPH were similar regarding to the three steps of decomposition (**Figures 5A–B**’). TG curves presented at least three distinct mass loss events with the final residue representing the ash content. In both cases, the maximum burning rate was at 320°C and the initial mass loss, which is related to the moisture release, occurred below 100°C. The next step, between 200 and 350°C, is related to the main mass loss stage and it corresponds to the hemicellulose, cellulose, and lignin decomposition. The third step, above 350°C for the *in natura* bagasse and around 300°C for the EPH, can be understood as a result of the final lignin degradation. In addition, the enzymatically pretreated bagasse exhibited a higher mass loss in this final lignin degradation step.

Thermo gravimetric/DTG curves for APH (**Figures 5C,C’**) showed a set of five mass loss events (between 4.8 and 23%) and DTG peak intensity completely different from the other treatments (**Table 1**). Also, the APH exhibited a residual mass of 45% (**Table 1**), which probably is the residual content of NaOH.

**Table 1:** Classification of the thermal decomposition events for each treatment and *in natura* bagasse.

Treatment	Event	Temperature interval (°C)	Mass loss (%)
*In natura* bagasse	(1)	25–100	6
	(2)	230–350	86.9
	(3)	390–500	3.9
	**Ash**	550–800	**3.2**
Enzymatic treated bagasse	(1)	25–100	6.9
	(2)	225–340	59.5
	(3)	360–480	26.8
	**Ash**	550–800	**6.8**
NaOH treated bagasse	(1)	25–150	13
	(2)	150–350	23
	(3)	500–650	8
	(4)	650–700	4.8
	(5)	700–800	6.2
	**Ash**	800	**45**

*Note the huge difference among the residual content (ash) from the bagasse samples. The NaOH pretreated bagasse exhibited 45% of residual mass, what may be residual NaOH deposited on the cellulosic matrix left after the pretreatment.*

Comparing the results of **Figure 5** and the previously described **Figure 3**, the **Figure 5** shows that the enzymatic treatment causes changes in the structure of the lignocellulosic material, but the NaOH treatment causes even more severe changes. On the other hand, **Figure 3** shows that the amount of reducing sugar was between 10 and 20 μmols for the sugar cane bagasse submitted to enzymatic treatment, but the pretreatment with NaOH produced even higher amount (over 40 μmols) of reducing sugar, i.e., the hydrolysis with NaOH is even greater. Thus, the results presented in **Figures 3** and **5** are in agreement and with complementary information for understanding the treatments efficiency.

### Cellulose Exposition Estimation

**FIGURE 6:**
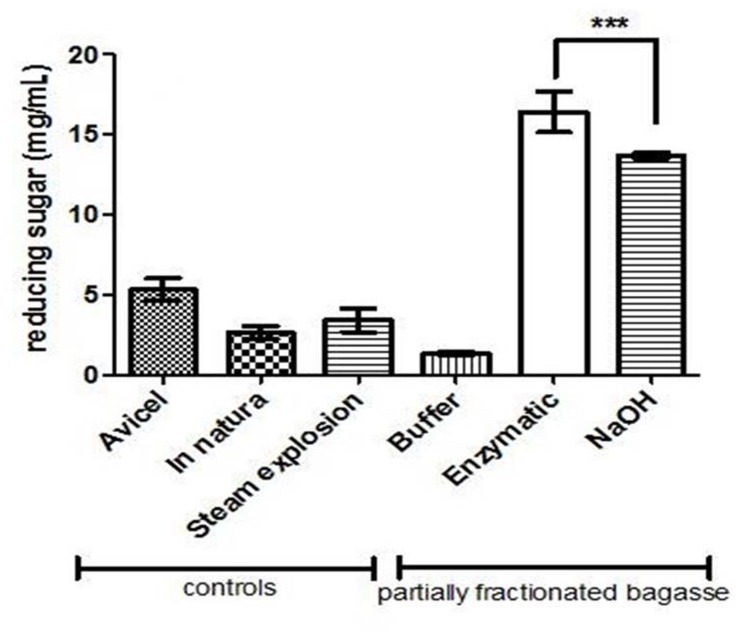
**Saccharification of enzymatic- and NaOH-pretreated holocellulose from sugarcane bagasse.** The assays were carried out using endoglucanase (2.7 U/g bagasse) and cellobiohydrolase (5.86 U/g bagasse) in 50 mM sodium acetate buffer, pH 5.0, for 24 h under stirring, and the reducing sugar quantified by DNS. Avicel was used as cellulases positive control. Steam explosion and *in natura* bagasse were used as pretreatment control. “Buffer” sample is the enzymatic pretreatment control, where buffer (sodium acetate) replaced the enzymes. The HSB was first pretreated with 50 mM ammonium acetate (buffer), enzymatic cocktail or NaOH. After that, the three pretreated material were hydrolyzed by cellulases. ^∗∗∗^ indicates statistically significance.

The sugar cane bagasse treated with the enzymatic consortium (endo-1,4-beta-xylanase, endo-1,5-α-L-arabinanase, α-L- AbfA, and xyloglucan endo-beta-1,4-glucanase) and with the alkali treatment were submitted to incubation with cellulases of *S. thermophilum*. As previously described, the NaOH treatment was more drastic, since completely broke the cellulose fibers and become its somehow inaccessible for later hydrolysis due to the residual NaOH present on the cellulosic mass. Then, when the material was submitted saccharification with cellulase, and the hydrolysis of EPH resulted in 21% more reducing sugar released than in the APH and also five times more than *in natura* bagasse (*p* < 0.0001 and *R*2 = 0.9906; **Figure 6**).

## Discussion

At a first glance, the NaOH pretreatment seemed to be the best choice because it removed more hemicellulose than the enzymatic pretreatment. APH exhibited morphological changes more significant than EPH and TG/DTG curves for the APH showed the complete disruption of the lignocellulosic structure. However, the complete removal of hemicellulose followed by profound morphological and structural changes in the biomass did not imply in higher cellulose hydrolysis rates. The best treatment would expose the cellulose for the following processes (e.g., saccharification) without producing harmful byproducts, regardless of how significant the physical modifications were.

That is why the hydrolysis of EPH was the highest in the CEE. The presence of hemicellulose and lignin in the *in natura* bagasse represented a significant obstacle for the cellulases explaining the low hydrolysis yield for this sample. The steam exploded bagasse and the Avicel samples exhibited lower hydrolysis yields, when compared to EPH, probably because of the remaining hemicellulose and the high crystallinity, respectively (Hall et al., 2010; Brodeur et al., 2011).

Alkali pretreatment processes have advantages as the use of lower temperatures than in other pretreatment technologies as well as the low degradation of complex sugars (Chen and Kuo, 2010). Nonetheless, the TG and Differential Thermal Analyses (TG/DTA) and the CEE applied in this work were able to show some flaws of the NaOH pretreatment. The 45% of residual mass pointed out in the NaOH treatment thermal profile might be residual NaOH (even after several washes with distilled water) that possibly inhibits the activity of cellulases explaining its lower hydrolysis rate compared to the enzymatic treatment.

That is why applying a core set of hemicellulases to replace chemical pretreatment is an alternative. Replacing hazardous chemicals by environment-friendly enzymes provides a mild biomass deconstruction allowing the following steps (saccharification and fermentation) to happen with no need for detoxification and/or recovery of byproducts. Unfortunately, the feedstock pretreatments reported in the literature so far have focused on chemical pretreatments (Kumar et al., 2009), but the improvement of heterologous expression systems has contributed to make enzymes more cost competitive (Zhu and Pan, 2010) encouraging their use.

Fungi have been extensively applied in the expression of heterologous enzymes (Adrio and Demain, 2003), but fungal co-cultivation in the biomass conversion is still incipient. The few studies about fungal co-cultivation (aiming biomass conversion) are only about white-rot fungi (Koroleva et al., 2002; Chi et al., 2007) with few exceptions using *Trichoderma reesei*, *A. niger*, *A. phoenicis*, and *A. oryzae* mostly dating from late 1990s (Dueñas et al., 1995; Gutierrez-Correa and Tengerdy, 1998; Gutierrez-Correa et al., 1999; Hu et al., 2011). Even though these studies have successfully applied fungal co-cultivation for biomass degradation, very little (if nothing) has been done afterward especially with recombinant strains. In fact, co-cultivation and molecular approaches (as heterologous protein expression) have so far been treated only as two independent ways to improve bioconversion processes, but never combined together (Kumar et al., 2008; Dashtban et al., 2009).

## Conclusion

The co-culture of recombinant strains was a potential strategy to simultaneously produce target enzymes. In addition, the enzymatic cocktail produced by this strategy was able to remove the hemicellulose from the sugarcane bagasse and expose the cellulose fibers. Even though the chemical pretreatment (using NaOH) has removed higher amount of hemicellulose, the biomass structure was severely affected and the cellulose was somehow inaccessible for later hydrolysis due to the residual NaOH present on the cellulosic mass. On the other hand, the enzymatic method released a minor amount of hemicellulose, but it was more efficient in exposing the cellulose for the saccharification.

Therefore, this is an efficient enzymatic process of hemicellulose removal, which can be applied on systems for bioethanol production. Further investigation will decode the simultaneous growth kinetics of microorganisms in co-culture conditions followed by scaling-up of the process that can be operated in industry.

## Author Contributions

ML and AD designed part of the molecular and biochemical experiments. AD and FS contributed in the molecular experiments producing the *A. nidulans* recombinant strains and its enzymatic cocktail. PC performed the Thermogravimetric and DTA. MP carried out the SEM. AS performed the sugarcane bagasse cell wall fractionation. JS purified the cellulases used in the saccharification. JJ and RL designed some experiments and contributed with final written. MP designed part of the experiments, contributed with final written and supervised all study. All authors contributed in the writing of the manuscript, read it and approved the final version of it.

## Conflict of Interest Statement

The authors declare that the research was conducted in the absence of any commercial or financial relationships that could be construed as a potential conflict of interest.

## Footnotes

1 Unicadata: http://www.unicadata.com.br/historico-de-producao-e-moagem.php?idMn=32&tipoHistorico=4&acao=visualizar&idTabela=1618&safra=2014%2F2015&estado=RS%2CSC%2CPR%2CSP%2CRJ%2CMG%2CES%2CMS%2CMT%2CGO%2CDF%2CBA%2CSE%2CAL%2CPE%2CPB%2CRN%2CCE%2CPI%2CMA%2CTO%2CPA%2CAP%2CRO%2CAM%2CAC%2CRR

## Abbreviations

AbfA: Arabinofuranosidase
AbnA: Endo-1,5-Arabinanase
AFEX: ammonia fiber expansion
ANOVA: analysis of variance
APH: alkali-pretreated holocellulose
CBS: centraalbureau voor schimmelcultures
CEE: cellulose exposition estimation
DNA: deoxyribonucleic acid
DTA: differential thermal analysis
DTG: derivative thermogravimetric analysis
DMSO: dimethyl sulfoxide
DNS: 3,5-dinitrosalicylic acid
EPH: enzymatic-pretreated holocellulose
FGSC: fungal genetics stock center
HBS: holocellulase from sugarcane bagasse
MM: minimal medium
NotI: notI restriction enzyme
PCR: polymerase chain reaction
SDS-PAGE: sodium dodecyl sulfate – polyAcrylamide gel electrophoresis
SEM: scanning electron microscopy
TG: thermogravimetric analysis
XbaI: XbaI restriction enzyme
XegA: xyloglucan specific endo-β-1,4-glucanase
XlnA: endo-1,4-xylanase.
